# Dog ownership and all-cause mortality in a population cohort in Norway: The HUNT study

**DOI:** 10.1371/journal.pone.0179832

**Published:** 2017-06-29

**Authors:** Magnhild Oust Torske, Steinar Krokstad, Emmanuel Stamatakis, Adrian Bauman

**Affiliations:** 1 HUNT Research Centre, Department of Public Health and Nursing, Faculty of Medicine and Health Sciences, NTNU—Norwegian University of Science and Technology, Levanger, Norway; 2 Levanger Hospital, Nord-Trøndelag Hospital Trust, Levanger, Norway; 3 Prevention Research Collaboration, Sydney School of Public Health, The University of Sydney, Camperdown, NSW, Australia; McMaster University, CANADA

## Abstract

**Objective:**

There has been increased interest in human-animal interactions and their possible effects on human health. Some of this research has focused on human physical activity levels, mediated through increased dog walking. Much of the reported research has been cross sectional, and very few epidemiological studies have examined the association between dog ownership and mortality in populations.

**Methods:**

We used data from the Norwegian county population-based Nord-Trøndelag HUNT Study (HUNT2, 1995–1997). Cox proportional hazards models were fitted to analyse the relationship between dog ownership and all-cause mortality. The median follow-up time was 18.5 years and the maximum follow-up time was 19.7 years.

**Results:**

In this population, dog owners were no more physically active than non-dog owners, both groups reporting a total of just over 3 hours/week of light and vigorous activity. Dog owners (n = 25,031, with 1,587 deaths during follow-up; 504,017 person-years of time at risk) had virtually the same hazard of dying as non-dog owners (Hazard ratio 1.00, 95% CI 0.91–1.09).

**Conclusions:**

We found no evidence for an association between the presence of a dog in the household and all-cause mortality or physical activity levels in this Norwegian population. Further epidemiological research is needed to clarify this relationship, as methodological limitations and an active Norwegian population sample means that generalizable evidence is not yet clear on dog ownership and mortality.

## Introduction

There has been an increase in research into human-animal interaction, and its possible effect on human health. A recent trend in this literature has focused on the potential benefits of dog ownership on increased human physical activity levels, mediated through dog walking. The American Heart Association has released a statement indicating the need for more data on the influence of pet ownership on the presence and reduction of cardiovascular disease risk factors and cardiovascular disease risk [1].

A rapid systematic review identified the study types in this field [see Text Box 1], with almost 60% being cross-sectional designs, examining the prevalence and correlates of dog walking. Dog walkers generally report more physically activity than dog owners who do not walk their dogs, or non-dog owners [2–4]. In a systematic review of dog walking, dog owners were more likely to meet physical activity guidelines through walking [5]. Around two thirds of dog owners walked their dogs, with a median of 160 minutes walked per week[5], and several studies suggested an hour more physical activity per week among dog owners who walked their dogs, compared to non-dog owners [2,4]. Nonetheless, most of the 29 studies in this systematic review were cross-sectional associations between dog ownership and walking or physical activity levels [5]. The magnitude of this effect was replicated in small-scale dog walking intervention trials [6,7]. However, some studies found the absolute difference in reported walking or physical activity between dog owners and non-dog owners to be small in magnitude or non-significant [8–10].

**Text Box 1. A rapid systematic review of the available literature on the association between dog ownership and health.**

A rapid systematic review was undertaken to profile papers in the published literature in this field to broadly classify papers on dogs and physical activity. The Scopus data base was used from 2000 to the end of 2016, and all papers with the Title terms (dog* AND (physical activity) or walk*)) were included. Two raters (SK and AB) considered all abstracts and coded them as [1] correlates or prevalence studies using cross sectional designs [including the association between dog walking and physical activity]; [2] editorials, opinion pieces; [3] measurement studies; [4] qualitative research; [5] interventions to increase DW; [6] epidemiological studies, longitudinal, cohorts, and [7] health benefits of pets, dogs. Inter-rater agreement was 87%, and all discrepancies were reconciled. Of the 194 papers available for review, 104 were not relevant to this study, as they were research on veterinary (canine) health or physiology. Of the remaining 90 papers, 58% were correlates or prevalence papers, 10% were editorials or opinion pieces, 9% were measurement studies, 7% were qualitative, 8% were interventions, 3% were epidemiological studies and 6% were about health benefits. Note that six measurement studies were excluded as they assessed accelerometry in dogs, and only human physical activity measurement studies were included.

The potential health benefits of dog ownership have been described for several decades, and thought to result from dog-human interactions, companionship and interaction, especially among older adults [11–14]. It is presumed that this social support leads to reduced loneliness and improved cardiovascular health, possibly partly mediated through reductions in blood pressure [15] or via stress reduction [11,16], possibly through oxytocin effects [17]. Two studies analysed the population-based Norwegian Health Study of Nord-Trøndelag (HUNT) cohort data using cross sectional analyses, and observed that cat owners were more anxious than dog owners, and incidentally reported that cat owners also reported higher blood pressure and body mass index measures, but did not make specific comparisons between dog owners and non-pet owners [18,19].

In summary, most of the evidence for health effects comes from cross-sectional studies, with associations noted between mental health indicators or blood pressure levels and pet (dog) ownership [15,20]. A recent review noted the frequency of these cross-sectional associations, and that the associations were attenuated when adjustments for age and socio-economic status were included [20].

There were no epidemiological studies of dog ownership and mortality directly [See Text Box 1], but pet ownership has been studied in relation to mortality outcomes. One study[21] followed 11,394 American adults for an average of 8.5 years. Although dog owners were more physically active than non-dog owners, they showed no difference in all-cause mortality (adjusted hazard ratio (HR) 1.19, 0.97–1.47). A further study of the National Health and Nutrition Examination Survey (NHANES) cohort in the US followed 3964 adults [22]. This study identified a 31% lower risk of coronary heart disease deaths and a 46% lower risk of stroke mortality among pet owners, but this was mostly attributable to cat ownership; dog owners showed non-significant reductions in risk, with HRs 0.82 and 0.76 for heart disease and stroke mortality respectively. One further study, also using the NHANES data found that previous cat ownership was associated with a reduced risk of myocardial infarction, but other pets or dog ownership was not studied [23]. No other pet or dog ownership and longitudinal population health outcome studies were identified.

The present paper explores the association between dog ownership and all-cause mortality in a Norwegian population-based cohort. We hypothesised that if adults have a dog, and walk that dog in the long term, the increased physical activity levels and possibly reduced stress levels may influence mortality outcomes.

## Methods

We used data from the second wave of the Nord-Trøndelag HUNT Study [24] (HUNT2, 1995–1997) in our study. All residents of Nord-Trøndelag County, Norway, aged 20 and above, were invited by mail. Data on the study participants was gathered using questionnaires, as well as clinical measurements. The participation rate in HUNT2 was 71.2% [25]. Written informed consent was obtained from all HUNT2 participants.

### Selection of study participants and primary measure used

**Fig 1:**
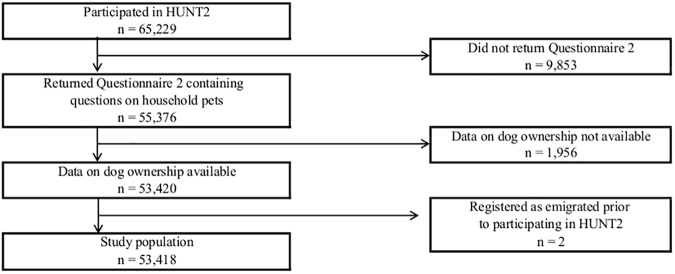
Selection of study participants. The Nord-Trøndelag HUNT Study HUNT2 (1995–1997).

The selection of study participants is shown in Fig 1. The base population were the 65,229 participants who had answered the first questionnaire (Q1) of HUNT2. Q1 was sent by mail along with the invitation, and was handed in at the time of participation. Questionnaire 2 (Q2) was handed out at the time of participation and was to be completed at home and returned by mail in a prepaid envelope [25]. The questions on household pets were in Q2, and we therefore excluded the 9,853 HUNT2 participants who had not returned Q2. The questions on household pets were asked in the context of household living conditions. The question of dog ownership was “Is there a dog in your home?” We excluded 1,956 participants who had not answered the question on whether or not there was a dog in the household. Finally, we excluded two study participants who according to the National Registry emigrated prior to their participation in HUNT2, as mortality data may not be available. The final sample consisted of 53,418 study participants.

### Covariates

Weight and height were measured at the time of participation in the HUNT2 survey, and BMI was calculated on the basis of these two measurements. All the other covariates were based on self-report. The highest level of education completed was measured using the categories “primary school”, “secondary school graduate” and “college/university graduate”. Marital state was measured using the following categories: “unmarried”, “married”, “widow, widower” and “divorced or separated”. Daily smoking was measured as a dichotomous variable (current smoker or non-smoker). We used the Hospital Anxiety and Depression Scale (HADS) to measure symptoms of anxiety and depression [26], and used a cut-off of ≥8 [27] to identify a high level of symptoms of anxiety and depression. Low intensity and high intensity physical activity were both measured per week in the last year, with the categories “None,” “Less than 1 hour,” “1–2 hours,” and “3 hours or more” [28].

### Ethical approval

The HUNT Study was approved by the Norwegian Data Inspectorate and the Regional Committee for Medical Research Ethics (REK Central). All participants in HUNT2 provided written informed consent. The present study was approved by the Regional Committee for Medical and Health Research Ethics (REC Central) (2015/1480).

### Statistical methods

We used Cox proportional hazards models to analyse the relationship between dog ownership and survival. The start of follow-up was participation in HUNT2, and the end of follow-up was April 2015. The median follow-up time was 18.5 years and the maximum follow-up time was 19.7 years. The HUNT database is regularly updated through the National Registry on dates of death and emigration of HUNT participants. We used the date of death as the endpoint. To protect the anonymity of study participants, all study participants who died in a certain month were registered as dying on the 15^th^ of that month. There were 12,698 deaths (23.8% of the study population) during follow-up. In total 208 study participants (0.4%) emigrated during follow-up, and they were censored at the time of emigration. We used attained age as the time scale, and adjusted for sex. We used the log-rank test and log-minus-log plots to test the proportional hazards assumption on the model.

#### Total cohort analysis

We estimated the hazard ratio of death in dog owners (n = 10,668) compared to the reference group of people who did not own a dog (n = 42,750). We considered age and sex to be confounders in the relationship between dog ownership and mortality. There was no missing data for sex and age. We adjusted for sex as a categorical variable. We used date of birth as the time scale in the Cox proportional hazards model, which may lead to a more effective control of age than including age as a variable in the model.[29]

#### Analyses on sub-cohort without missing data

To explore the relationship between dog ownership and mortality, we adjusted for confounders as well as possible mediators in three models. However, with the exception of sex and age, a considerable part of the cohort had missing data on one or more of the covariates used in the models. In order to perform all these three analyses on the same sample, we identified a sub-cohort consisting of “complete cases” only (n = 28,746), who did not have missing on any of the covariates.

Model 1 was adjusted for sex and age only, as previously described in the total cohort analysis section.

Model 2 was adjusted for the variable in Model 1 in addition to education and marital status, which we considered to be possible confounders in the relationship between dog ownership and mortality.

In Model 3, we adjusted for the variables in Model 2, as well as a set of variables related to health and lifestyle factors. These variables were: High levels of symptoms of anxiety and depression, BMI, smoking and physical activity. Several of the variables in Model 3 may be mediators in the relationship between dog ownership and mortality. Low intensity and high intensity physical activity per week in the last year were entered into the model as two separate categorical variables. Daily smoking was entered as a dichotomous variable (smoker/non-smoker). As there was evidence that the relation between weight and mortality was not linear, BMI was entered as a categorical variable with five categories (<18.5, 18.5–24.9, 25–29.9, 30–34.9, and ≥35). A Kaplan-Meier plot suggested that there might be a very slight difference in survival in younger versus older dog owners. We therefore stratified by age (below and above the age of 65) and repeated the analyses on the complete cases cohort. We also stratified by sex. Analyses were performed using Stata 13.1.

## Results

General characteristics of the study population are showed in Table 1. The mean age of dog owners was 5.4 years lower than non-dog owners. A higher proportion of dog owners were smokers than in people who did not own a dog, but there was little difference between the two groups in light and vigorous physical activity and mean scores for anxiety or depression symptoms.

**Table 1:** Characteristics of the study participants.

			Dog owners				Not dog owners		
		n	%	Mean	SD	n	%	Mean	SD
N		10,668				42,750			
Age		10,688		46.7	14.1	42,750		51.2	17.7
Females		5,633	52.8			23,306	54.5		
Deaths during follow-up		1,586	14.9			11,112	26.0		
Emigration during follow-up		45	0.4			161	0.4		
Marital status	*Single*	2,294	21.5			10,193	23.9		
	*Married*	7,226	67.9			25,485	59.8		
	*Divorced/separated*	697	6.5			2,876	6.7		
	*Widow/widower*	432	4.1			4,096	9.6		
Education	*Primary school*	3,454	33.4			14,848	36.6		
	*Secondary school*	4,870	47.1			17,224	42.4		
	*University/college*	2,016	19.5			8,524	21.0		
***Health***	*** ***				** **				** **
Self-reported health "poor" or "not so good"^a^		2,625	24.8			11,735	27.7		
Low quality of life^b^		306	2.91			1032	2.46		
Mean anxiety score (HADS-A)		9,272		4.3	3.3	35,468		4.1	3.2
Mean depression score (HADS-D)		9,922		3.5	3.1	38,850		3.4	3.0
***Health-related behavior***					** **				** **
Daily smoker		3,270	32.1			10,738	26.5		
Possible alcohol problem^c^		748	8.4			2,553	8.5		
Hours of low intensity physical activity per week in the last year		9,206		2.1	0.9	35,888		2.0	0.9
Hours of vigorous physical activity per week in the last year		7,789		1.2	1.1	29,972		1.1	1.0
***Measurements***					** **				** **
BMI		10,638		26.4	4.1	42,455		26.3	4.1

The Nord-Trøndelag Health Study (HUNT2), 1995–97.

^a^ As reported on the stand-alone question “How is your health at the moment?”. The other possible response categories were “good” and “very good”.

^b^ Reporting that they were "somewhat dissatisfied", "dissatisfied" or "very dissatisfied" with life on a stand-alone question on quality of life (“Thinking about your life at the moment, would you say that you by and large are satisfied with life, or are you mostly dissatisfied?”)

^c^ CAGE (Cut down, Annoyed, Guilty, Eye-opener) score > = 2 (Ewing JA. Detecting alcoholism. The CAGE questionnaire. JAMA. 1984 12;252(14):1905–7)

### Total cohort

We estimated the hazard ratio (HR) of death in the total sample (n = 53,418). Using people who did not own a dog as the reference group, dog owners had a hazard ratio (HR) for all-cause mortality of 0.98 (95% confidence interval (CI): 0.93–1.03), adjusted for sex and age. There were 12,698 deaths during follow-up, and the person-time at risk was 892,985 person-years.

### Sub-cohort without missing data

The hazard ratios adjusted for possible confounders are shown in Table 2. There were 28,746 study participants in the cohort without missing data for any of the study variables. The number of deaths in the cohort was 4,233, and the person-time at risk was 504,017 person-years. The hazard ratios of death were very similar in dog owners and non-dog owners, and adjustment changed the estimates only marginally.

**Table 2:** The hazard ratios of death in dog owners compared to people who do not own a dog.

		Model 1		Model 2		Model 3	
		HR	95% CI	HR	95% CI	HR	95% CI
**Whole sample**		1	·	1	·	1	·
	Others (reference)						
	Dog in the household	0.99	0.91–1.09	1	0.91–1.09	1	0.91–1.09
**Sample stratified by age**							
	**Age <65**	1	·	1	·	1	·
	Others (reference)						
	Dog in the household	0.99	0.88–1.12	1	0.89–1.13	1	0.88–1.12
	**Age 65+**	1	·	1	·	1	·
	Others (reference)						
	Dog in the household	1.01	0.89–1.15	1.01	0.89–1.15	1.02	0.89–1.16
**Sample stratified by sex**		** **		** **	** **	** **	** **
	**Men**						
	Others (reference)	1	·	1	·	1	·
	Dog in the household	0.96	0.86–1.08	0.96	0.86–1.07	0.98	0.88–1.09
	**Women**	1	·	1	·	1	·
	Others (reference)						
	Dog in the household	1.04	0.90–1.21	1.05	0.91–1.22	1.02	0.88–1.18

The Nord-Trøndelag Health Study (HUNT2, 1995–97). Follow-up from the time of participation in HUNT2 until April 2015.Model 1: Adjusted for sex and age. Model 2: Model 1 + adjusted for education and marital statusModel 3: Adjusting for all above variables and anxiety and depression scores, BMI, physical activity levels and smoking.

### Stratified analyses

Results of survival analyses stratified by age (>65 years and ≤65 years) are shown in the middle panel of Table 2. Younger dog owners (n = 25,031, 1,587 deaths during follow-up, time at risk: 457,983 person-years) had virtually the same hazard of dying as non-dog owners, whereas the hazard ratios in dog owners in the oldest age group (n = 3,715, 2,646 deaths during follow-up, time at risk: 46,033 person-years) were very slightly higher than the reference group.

Results of analyses stratified by sex are shown in the lower panel of Table 2. In women (n = 14,868, 1,708 deaths during follow-up, time at risk: 264,918 person-years) dog owners had a very slightly higher hazard of death than the reference group. In men (n = 13,878, 2,525 deaths during follow-up, time at risk: 239,098 person-years)), the hazard ratio for dog owners was very slightly lower than in the reference group.

## Discussion

While dog ownership is widely believed to confer health benefits, there is a paucity of long-term prospective studies. Our study is one of the few epidemiological studies that examined the long-term outcomes of dog ownership with mortality risk in a large population-based cohort. We found that living in a house with a dog was not associated with mortality, and this finding persisted in sex and age sub-group analyses. Further, participants in this study living in households with dogs did not appear to be more physically active than participants who did not.

### Other studies with mortality outcomes

Although the epidemiological literature on dog presence and mortality is limited, findings are consistent. Our results are in agreement with US studies that utilized NHANES data that failed to show any significant association between dog ownership and mortality risk [21,22]. There was some protective association noted among current and previous cat owners [22,23] but this was different to the cross-sectional studies suggesting cat owners were at higher risk for depression and for hypertension [18,19].

### Limitations of the literature as a whole

There are at least two possible explanations for the consistent, but limited association between dog ownership and mortality. Owning a dog may have health benefits [1,11–14], but these benefits may not be not translated into reduced mortality or a longer life. However, studies in the field suffer from methodological drawbacks that are important to consider when interpreting their findings. None of the studies to date included questions on dog presence/ownership for the purpose of studying human health. For example in the NHANES-II study, dog ownership questions were asked in relation to allergic disease [23]. In NHANES-3 [21,22]and in the HUNT Study [18,19] the questions were about presence of a dog in the household, with no further details about actual “ownership”, including who (if anyone) walks the dog, and the amount of interaction with the dog. Further, exposure to dogs was usually measured only once at baseline, and not assessed on subsequent occasions. For these reasons, the household dog ownership questions may not detect associations with prospective health outcomes. Another universal limitation of the literature is the lack of information about the age, breed, and timing of dog acquisition that could be used to estimate length of the dog’s life in relation to the timing of mortality events. As average life of a mid-size dog is approximately 11–13 years [30], the large majority of HUNT participants living in houses with dogs (mean age under 47 years) would have outlived their dogs, but it is unknown if or when another dog was acquired. The absence of such information may add non-specific error to any observed association between exposure and outcomes and further attenuate mortality associations to the null.

Regardless of the likely explanation of the null findings, it is possible that the limited literature on this question is a sign of publication bias, i.e. other researchers have carried out similar analyses and because they have also found null associations, the data were never published. This also indicates the limitations of the plethora of cross-sectional evidence, which provides an optimistic potential for dog ownership and health, that needs to be confirmed by repeated longitudinal epidemiological evidence.

### Dog walking and physical activity

The findings in this study were somewhat surprising, given the reasonably consistent evidence from systematic reviews that dog owners are more physically active than non-dog owners [3,5]. Increased physical activity through dog walking is the key proposed mechanism for the postulated long term health benefits of dog ownership but none of the mortality studies above or the HUNT cohort included *specific questions* on dog walking. We found no differences in physical activity between participants with and without dogs and this is in agreement with only a few studies [8–10], but may reflect this generally more active rural Norwegian population.

Further, characteristics of the HUNT cohort may have contributed to the results we found in our material. The Nord-Trøndelag region is mostly rural, and a sizeable proportion of household dogs may have been “working dogs” used for hunting or dog sledding, not as family pets promoting regular dog walking. Moreover, the study population is characterized by a relative high level of physical activity, so dog ownership may not have any additional effect on the level of activity. By contrast, dog ownership may increase physical activity in cities and urban areas where opportunities for other outdoor activities are more limited.

### Strengths and weaknesses

This study of dog ownership and mortality has the longest follow-up to date although this could be seen as both a strength and a weakness due to relatively short lifespan of dogs and the absence of information on subsequent dog ownership, as discussed above. Our analyses were statistically powerful and utilized a large cohort with a high response rate and over 12,000 events. We used an objective registry data to measure the endpoint, and the only source of loss to follow-up was emigration, which was very low at 0.4%. Like the remaining studies in the field [21,22], the main limitation of the study was the lack of specificity of the dog ownership questions, which did not allow us to infer dog caring roles, dog walking behaviours or duration of dog in the household exposure, both prior to and after study participation. We had no data specifically on dog walking per se, so we do not know whether dog owners walked their dogs but compensated with less discretionary physical activity time. Finally, substantial original data were excluded from multivariate analyses due to missing data in at least one variable, although we have no evidence that any systematic bias was introduced.

## Conclusions

We found no evidence for an association between the presence of a dog in the household and all-cause mortality or physical activity in this large regional cohort from Norway. The methodological limitations of our study are endemic in this field of research and as such our work can neither confirm nor refute that dog ownership is beneficial for mortality risk, especially not in urban areas. This question needs to be answered in future studies that, ideally, are designed for the purpose and collect additional information on dog ownership, dog walking and dog survival. Until we have such data, dog ownership can be promoted only as a modality that may improve quality of life and aspects of health other than reduced mortality risk.
